# The Impact of a 12‐Month Exercise Intervention on Cognitive Function in Adults with Down Syndrome

**DOI:** 10.1002/alz.086292

**Published:** 2025-01-09

**Authors:** Lauren Ptomey, Brian Helsel, Jessica Danon, Amanda Szabo‐Reed, Richard Washburn, Joseph Donnelly

**Affiliations:** ^1^ University of Kansas Medical Center, Kansas City, KS USA; ^2^ University of Kansas Alzheimer’s Disease Research Center, Fairway, KS USA

## Abstract

**Background:**

Evidence in adults without Down syndrome (DS) suggests that exercise during mid‐life improves cognitive function and decreases risk of later life dementia. Studies supporting this relationship in adults with DS are limited. The purpose of this study was to examine changes in cognitive function after a 12‐mo exercise intervention in adults with DS without dementia.

**Method:**

Adults with DS were randomized (2:2:1) to one of three exercise interventions: remotely delivered group exercise sessions conducted 1x/wk. (RL), remotely delivered group exercise sessions conducted 3x/wk. (RH), or a usual care control (UC) which did not receive the remotely delivered sessions. All groups received an activity tracker, access to resources for increasing exercise, and twice monthly 20‐min remotely delivered individual support/education sessions. Physical activity (accelerometry) was assessed across 12 mos. Cognitive function was assessed at baseline, 6, and 12 months, using the CANTAB^®^ DS Battery, which included tests measuring multitasking, episodic memory, and reaction time. Linear mixed‐effects analyses were used to test the effect of the intervention on changes in cognitive function, controlling for age, sex, and level of intellectual disability

**Result:**

Eighty‐one adults with DS (age ∼27yrs., ∼55% female, ∼24% minorities) were randomized to the RH (n = 34), RL (n = 32) or UC (n = 15) groups. Study retention was 100% and study adherence to the remote exercise sessions was 90% in the RH arm and 79% in the RL arm. MVPA increased by 10.6 min/day and 1.4 mins/day in the RH and UC arms, respectively, but decreased by ‐3.2 mins/day in the RL arm. Across all groups episodic memory (p = 0.018), 5‐choice movement time (p = 0.017), and 5‐choice reaction time (p = 0.022) improved across the intervention, however, there were no significant group or group x time effects.

**Conclusion:**

In adults with DS, participation in a 12‐month exercise intervention led to improvements in episodic memory and reaction time regardless of intervention arm. Future adequately powered trials are warranted to examine the impact of long‐term exercise on cognition function outcomes compared to a non‐exercise control.

